# Supplementary material for: Self-esteem and stress: a structural equation modelling of biosocial determinants, psychological mediators and anxiety among Malaysian undergraduates
Source: PeerJ. 2025 Jun 24;13:e19304. doi: 10.7717/peerj.19304 (PMC12204089; doi:10.7717/peerj.19304)
Supplement: Supplemental Information 1 [file peerj-13-19304-s001.pdf]

Table S1 Sub-analysis of problematic internet use, social support and mental health literacy based on domains

Social Factors	Domains	Mean (SD)
Problematic internet use	Control disorder	3.0 (0.8)
	Neglect	2.9 (0.8)
	Obsession	2.6 (0.9)
Social Support	Family	5.0 (1.3)
	Friends	4.9 (1.2)
	Significant other	5.1 (1.4)
Mental health literacy	Knowledge of mental health problems	4.1 (0.7)*
	Erroneous belief	4.6 (1.0)*
	Help-seeking and first aid skills	4.0 (1.0)*
	Self-help strategies	4.5 (1.0)*

Note: SD = Standard deviation; *Median (IQR)

Table S2 Sub-analysis of social support based on year of study

Year of study	Mean (SD)	ANOVA Test	
		F value	p-value
Year 1	59.2 (1.4)	2.524	0.039*
Year 2	60.4 (1.5)		
Year 3	61.4 (1.5)		
Year 4	62.3 (1.5)		
Year 5	63.2 (1.6)		

*Post hoc analysis: Year 1 undergraduates scored significantly lower than year 4 undergraduates (p=0.007).

Table S3 Sub-analysis of mental health literacy

Item	Question	Mean (SD)
**Knowledge**		4.25 (0.5)
MHL7	Changes in brain function may lead to the onset of mental disorders	3.99 (0.8)
MHL10	One of the symptoms of depression is the loss of interest or pleasure in most things	4.42 (0.6)
MHL11	The symptoms' length is one of the important criteria for the diagnosis of a mental disorder	4.13 (0.7)
MHL12	Mental disorders affect people's thoughts	4.41 (0.7)
MHL14	A person with schizophrenia may see and hear things that nobody else sees and hears	4.14 (0.8)
MHL15	Highly stressful situations may cause mental disorders	4.43 (0.6)

**Erroneous Belief**		4.40 (0.6)
MHL2R	Mental disorders don’t affect people’s behaviors	4.29 (0.9)
MHL5R	Mental disorders don’t affect people’s feelings	4.42 (0.8)
MHL6R	Only adults have mental disorders	4.51 (0.7)
**Help Seeking**		4.04 (0.7)
MHL4	If I had a mental disorder, I would seek for a psychologist’s help	3.87 (0.9)
MHL8	If someone close to me had a mental disorder, I would encourage her/him to see a psychiatrist	4.23 (0.7)
MHL16	If I had a mental disorder, I would seek for a psychiatrist’s help	4.03 (0.9)
**Self-Help**		4.45 (0.5)
MHL1	Physical exercise contributes to a good mental health	4.50 (0.6)
MHL3	Sleeping well contributes to a good mental health	4.53 (0.6)
MHL9	A balanced diet contributes to a good mental health	4.33 (0.7)
MHL13	Doing something enjoyable contributes to a good mental health	4.47 (0.6)
