# Supplementary material for: Self-esteem and stress: a structural equation modelling of biosocial determinants, psychological mediators and anxiety among Malaysian undergraduates
Source: PeerJ. 2025 Jun 24;13:e19304. doi: 10.7717/peerj.19304 (PMC12204089; doi:10.7717/peerj.19304)
Supplement: Supplemental Information 4 [file peerj-13-19304-s004.pdf]

Codebook for Categorical Data

	Categorical Data	Code
1	Gender	0 = Male<br>1 = Female
2	Ethnicity	0 = India<br>1 = Malay<br>2 = Chinese<br>3 = Others
3	Physical Activity	1 = Inactive , values less than 600<br>2 = Minimally active, values between 600 and 2999<br>3 = HEPA active, values more than 2999
4	Smoking	0 = No<br>1 = Yes
5	Alcohol	0 = No<br>1 = Yes
6	Illicit Drug	0 = No<br>1 = Yes
7	Problematic Internet Use	0 = Low, value less than 13<br>1 = High, value of 13 and above
8	Social Support	0 = Low, value less than 30<br>1 = Moderate, value between 30 and 60<br>2 = High, value more than 60
9	Self-esteem	0 = Low, value less 26<br>1 = Moderate, value between 26 and 29<br>2 = High, value more than 30
10	Stress	0 = Mild, value less than 14<br>1 = Moderate, value between 14 and 26<br>2 = Severe, value more than 26
11	Anxiety	0 = Minimal, value between 0 and 4<br>1 = Mild, value between 5 and 9<br>2 = Moderate, value between 10 and 14<br>3 = Severe, value more than 14
